# Aqueous humor heat-shock protein 70, periostin, and irisin levels in patients with pseudoexfoliation syndrome

**DOI:** 10.5935/0004-2749.20200046

**Published:** 2024-02-11

**Authors:** Mete Güler, Süleyman Aydın, Selma Urfalıoğlu, Meltem Yardım

**Affiliations:** 1 Department of Ophthalmology, School of Medicine, Kahramanmaraş Sütçü İmam University, Kahramanmaraş, Turkey; 2 Department of Biochemistry, School of Medicine, Fırat University, Elazığ, Turkey

**Keywords:** Aqueous humor, Irisin, HSP70, heat-shock proteins, Periostin, Pseudoexfoliation syndrome, Humor aquoso, Irisina, Proteínas de choque térmico HSP70, Periostina, Síndrome de pseudoexfoliação

## Abstract

**Purpose:**

To measure humor heat-shock protein 70, periostin, and irisin levels in patients with pseudoexfoliation syndrome and cataract (without glaucoma), and compare them with those of patients with cataract but without pseudoexfoliation.

**Methods:**

We examined 31 eyes of 31 patients with pseudoexfoliation and cataract (without glaucoma) and 30 eyes of 30 patients with cataract. We collected aqueous humor samples from all patients at the time of cataract surgery through a limbal paracentesis via a 25-gauge cannula mounted on a tuberculin syringe that received 100 to 150 µL of aqueous humor. We measured levels of aqueous humor Heat shock protein 70, periostin, and irisin using enzyme-linked immunosorbent assay methods.

**Results:**

The age (p=0.221) and gender (p=0.530) means were similar between the pseudoexfoliation and control groups. The mean Heat shock protein 70 level (29.22 ± 9.46 ng/mL; 17.88-74.46) in the pseudoexfoliation group was significantly higher than that in the control group (19.03 ± 7.05 ng/mL; 9.93-35.52; p<0.0001). The mean periostin level was significantly higher (6017.32 ± 1271.79 pg/mL; 3787.50-10803.57) in the pseu doexfoliation group than that in the control group (4073.63 ± 1422.79 pg/mL; 2110.44-7490.64; p<0.0001). The mean irisin level (53.77 ± 10.19 ng/mL; 29.46-71.16) was significantly higher than that in the control group (39.29 ± 13.58 ng/mL; 19.41-70.56; p<0.0001).

**Conclusions:**

Heat shock protein 70, periostin, and irisin levels increase in the aqueous humor of patients with pseudoexfoliation without glaucoma.

## Abstract (pt)

**Objetivo:**

Comparar os níveis de proteína de choque térmico 70, de periostina e de irisina no humor aquoso de pacientes com pseudoexfoliação com catarata sem glaucoma e compará-los com pacientes com catarata sem pseudoexfoliação.

**Métodos:**

Trinta e um olhos de 31 pacientes com pseudoexfoliação com catarata sem glaucoma e 30 olhos de 30 indivíduos com catarata foram incluídos neste estudo. Amostras de humor aquoso foram coletadas de todos os pacientes no momento da cirurgia de catarata e obtidas através de uma paracentese límbica por meio de uma cânula de calibre 25 acoplada a uma seringa com tuberculina. Foram coletados 100 a 150 µL de humor aquoso. Os níveis de proteína de choque térmico 70, de periostina e de irisina no humor aquoso foram medidos usando o método de ensaio imunossorvente ligado a enzima.

**Resultados:**

A média da idade (p=0,221) e sexo (p=0,530) foram semelhantes entre os grupos pseudoexfoliação e controle. Os níveis médios de proteína de choque térmico 70 foram 29,22 ± 9,46 ng/mL (17,88-74,46) e 19,03 ± 7,05 ng/ mL (9,93-35,52) nos grupos pseudoexfoliação e controle, respectivamente. Os níveis de proteína de choque térmico 70 foram maiores no grupo pseudoexfoliação (p<0,0001). O nível médio de periostina foi de 6017,32 ± 1271,79 pg/mL (3787,50-10803,57) no grupo pseudoexfoliação e 4073,63 ± 1422,79 pg/mL (2110,44-7490,64) no grupo controle. O nível médio de periostina também foi maior no grupo pseudoexfoliação (p<0,0001). Os níveis médios de irisina foram 53,77 ± 10,19 ng/mL (29,46-71,16) e 39,29 ± 13,58 ng/mL (19,41-70,56) nos grupos pseudoexfoliação e controle, respectivamente. O nível médio de irisina foi maior no grupo pseudoexfoliação do que no grupo controle (p<0,0001).

**Conclusões:**

Os níveis de proteína de choque térmico 70, de periostina e de irisina aumentam no humor aquoso de pacientes com pseudoexfoliação sem glaucoma.

## INTRODUCTION

Pseudoexfoliation (PEX) syndrome is characterized by the deposition of elastic microfibrillar material in the anterior segment of the eye and different tissues of the body, such as the skin and connective tissue portions of visceral organs^(1)^. The prevalence of PEX syndrome increases markedly with age^(2)^. Iris depigmentation leading to peri-pupillary transillumination defects, mild trab ecular meshwork hyperpigmentation, secondary open-angle glaucoma, and phacodonesis or lens subluxation caused by zonular dehiscence are some of the anterior segment manifestations of PEX^(3)^.

Heat-shock proteins (HSPs) have a wide range of functions, which include protecting against external stress and injury; and helping to regulate metabolism during normal development, differentiation, and growth^(4)^. Heat-shock protein 70 (HSP-70) is undetectable under normal conditions but is highly induced in cells experiencing stress^(5)^. Mushtaq et al. found that HSP-70 gene and protein expression were increased during the active phase of cell migration during corneal wound healing^(6)^.

Periostin is an extracellular matrix protein belonging to the fasciclin family that plays a role in the process of remodeling during tissue and organ development or repair^(7)^ by regulating cell adhesion, cell differentiation, and organization of extracellular matrix^(8)^. Qu et al. demonstrated that periostin is exclusively produced in the basal layer of human limbal epithelial cells of the cornea^(9)^. Periostin may have role in trabecular meshwork development^(10)^.

Irisin is a hormone-like molecule mainly released by skeletal and cardiac muscle cells in response to exercise. Irisin induces browning of the white adipose tissue and has been shown to regulate glucose and lipid homeostasis^(11)^ and reduce oxidative stresses and apoptosis^(12)^. Few ophthalmologic studies have focused on irisin, but serum and vitreous irisin concentrations have been found to be low in patients with proliferative diabetic retinopathy^(13)^.

The precise etiology and pathogenesis of PEX syndrome remain unknown^(14)^. In this study, we compared levels of aqueous humor HSP-70, periostin, and irisin in patients with PEX and cataract without glaucoma to those in patients with cataract without PEX. We aimed to identify molecules that may be associated with the pathogenesis of PEX syndrome. We included only patients without glaucoma in the study, to prevent confounding effects of glaucoma and increased intraocular pressure on the aqueous humor HSP-70, periostin, and irisin levels.

## METHODS

We followed the tenets of the Declaration of Helsinki; our institutional ethics committee approved the study (approval number, 2017/01/12), and we obtained informed consent from all of the participants. We examined aqueous humor from 31 eyes of 31 patients with PEX and cataract, and 30 eyes of 30 controls with cataract. We excluded patients with a history of diabetes mellitus, systemic arterial hypertension, systemic vasculopathies, retinal disease, ocular surgery, ocular trauma, and ocular inflammation. We determined the presence of clinical PEX based on slit-lamp examination findings showing fibrillin deposits on the anterior lens capsule and the pupillary margin. The patients with PEX had no history or signs of glaucoma. We performed complete ophthalmic examinations, including a review of the medical history, slit-lamp biomicroscopy of anterior and posterior segment, gonioscopy, Goldmann applanation tonometry (GAT), ultrasound pachymetry, and glaucoma scan protocols of spectral-domain OCT (Optovue RTVue-100, Optovue, Fremont, CA) to exclude glaucoma in patients with PEX. We obtained GAT measurements with patients in a sitting position and calculated the mean of three consecutive readings.

Surgeons collected 100- to 150-µL aqueous humor samples from all patients at the time of cataract surgery through a limbal paracentesis via a 25-gauge cannula mounted on a tuberculin syringe. Surgeons paid special attention to avoid potential blood contamination of the samples, which were stored at -80 °C until further processing. We diluted the aqueous humor samples 1:3 with phosphate-buffered saline (pH 7.4) to obtain adequate volumes for enzyme-linked immunosorbent assay (ELISA) tests. We used ELISA kits for aqueous humor HSP-70 (human HSP-70 catalog number, EH3242; Fine Biotech, Wuhan, China), periostin (human POSTN/OSF2 [periostin]; catalog number, EH0255; Fine Biotech, Wuhan, China), and irisin (human irisin catalog number,EH4702; Fine Biotech, Wuhan, China) according to the manufacturers’ protocols. First, we added HSP-70, periostin, or irisin to single wells coated with antibodies and incubated the reactions. We then added anti-HSP-70, anti-periostin, and anti-irisin antibodies labeled with biotin to combine with streptavidin-HRP and form an immune complex. We removed unbound enzymes by washing and determined specimen absorbance values on a ChroMate, Microplate Reader P4300 (Awareness Technology Ins truments, Palm City, FL, USA) at a wavelength of 450 nm. We multiplied the results by three for the analysis due to the initial 1:3 dilution with phosphate-buffered saline. We expressed values as ng/mL and pg/mL.

### Precision of the equipment and tests

We keep our laboratory equipment properly calibrated, functioning, and cleaned before biological sample analyses. We used prescribed clinical quality controls, including method validation, analytical characteristics [precision (within and between runs); accuracy (measured and certified values), and split sampling (results of split samples from two laboratories) to ensure that our measurements of HSP-70, periostin, and irisin in aqueous humor were analytically valid.

### Summary of assay validation

The coefficients of variance (CV) intra-assay (within the same day) and inter-assay (between days) were performed as described^(15)^, and we calculated CV values by dividing the standard deviation by the mean and multiplying the result by 100. The intra-assay CV, inter-assay CV, detection range, and sensitivity of the HSP-70 kit were 7%, 11%, 0.781-50 ng/mL, and <0.469 ng/mL, respectively. The intra-assay CV, inter-assay CV, detection range, and sensitivity of the periostin kit were 11%, 12%, 0.156-10 ng/mL, and <0.094 ng/mL, respectively. The intra-assay CV, inter-assay CV, detection range, and sensitivity of the irisin kit were 12%, 14%, 1.56-100 ng/mL and <0.938 ng/mL, respectively.

### Statistical analysis

We performed all statistical analyses using the Statistical Package for Social Sciences (SPSS version 17). We calculated descriptive statistics like means, standard deviations, and minimum-maximum values to report the data. We used independent samples t-tests to analyze the quantitative data and considered p<0.05 as statistically significant.

## RESULTS

**Figure 1:**
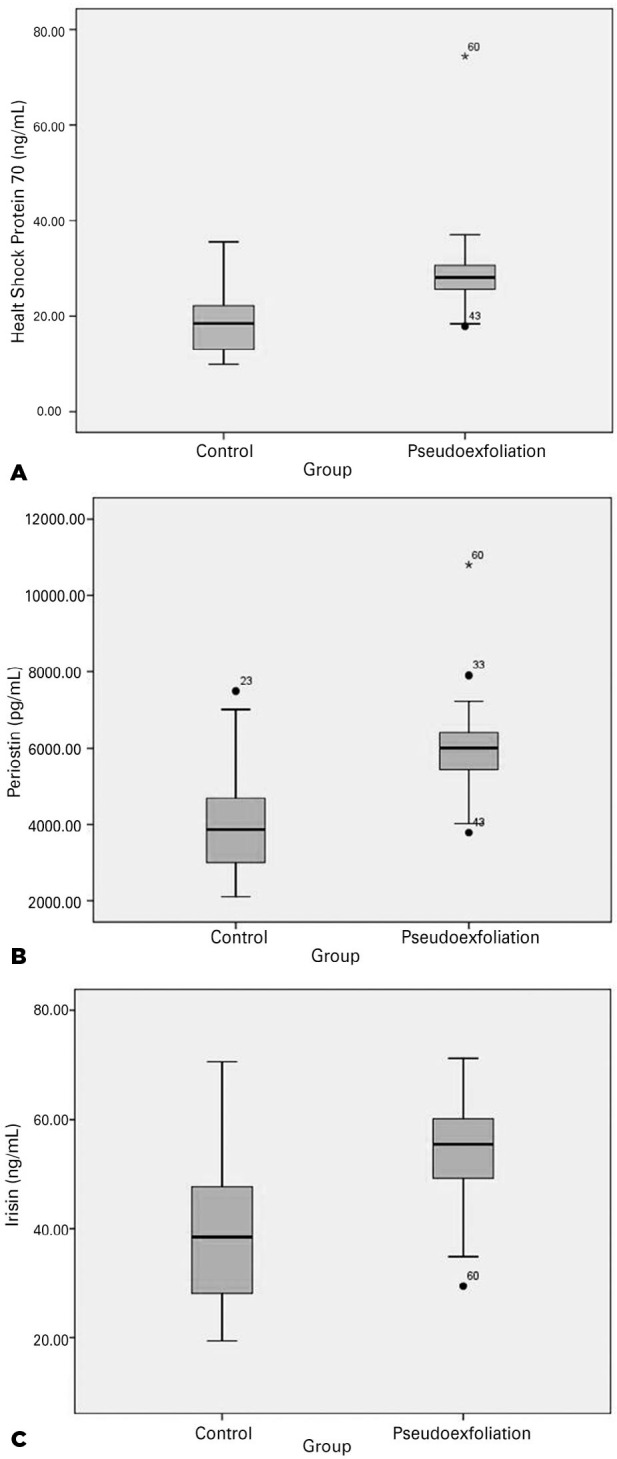
**A, B, C:** Box-plot graphics showing the distribution of heat-shock protein-70, periostin, and irisin levels in the two groups. The black lines in the diagrams show the median values for each group.

The mean age in the PEX group was 69.19 ± 8.01 years (57-83), and it was 66.50 ± 8.96 years (51-85) in the control group for a nonsignificant difference (p=0.221). In the PEX group, 19 patients were men, and 12 were women; in the control group, 16 were men, and 14 were women for a nonsignificant difference (p=0.530, Pearson’s chi-squared test). The mean HSP-70 levels were 29.22 ± 9.46 ng/mL (17.88-74.46) and 19.03 ± 7.05 ng/mL (9.93-35.52) in the PEX and control groups, respectively (Figure 1A). The HSP-70 levels were higher in the PEX group (p<0.0001) than in the control group. The mean periostin level was significantly higher (6017.32 ± 1271.79 pg/mL; 3787.50-10803.57) in the PEX group than in the control group (4073.63 ± 1422.79 pg/mL; 2110.44-7490.64; p<0.0001; Figure 1B). The mean irisin level was significantly higher in the PEX group (53.77 ± 10.19 ng/mL; 29.46-71.16) than in the control group (39.29 ± 13.58 ng/mL; 19.41-70.56; p<0.0001; Figure 1C).

## DISCUSSION

According to a Medline search, the levels of HSP-70, periostin, and irisin in the aqueous humor of patients with PEX have not been studied before. We found the levels of HSP-70, periostin, and irisin in the aqueous humor of patients with PEX to be approximately 1.5-fold higher than those in the controls without PEX. The exact reasons for these increments are unknown, but we believe that subclinical inflammation and oxidative stress may contribute to the increment of these substances in patients with PEX.

Inflammatory cytokines have been reported in PEX: Zenkel et al. reported that early stages of PEX syndrome were characterized by ~3-fold elevations of interleukin (IL)-6 and IL-8 levels in the aqueous humor with concomitant 2-fold increases in mRNA expression levels in the anterior segment tissues as compared with the levels of the same markers in controls^(16)^. Sarenac Vulovic et al. found increased aqueous humor levels of tumor necrosis factor (TNF)-α, IL-17, and IL-6 in the early and late stages of PEX and cases of PEX with glaucoma compared with the levels in a control group^(17)^. Oxidative stress is also involved in PEX. Dursun et al. investigated the oxidative stress status of the aqueous humor and serum of patients with PEX syndrome and PEX glaucoma. They measured total oxidative stress (TOS), total antioxidant capacity (TAC), paraoxonase (PON), and arylesterase (ARE) levels in aqueous humor and serum. TAC, PON and ARE levels in aqueous humor and serum of the PEX syndrome and PEX glaucoma patients were significantly decreased compared with control group. TOS values were higher in patients with PEX syndrome and PEX glaucoma than controls^(18)^. PON and ARE are both esterase with lipophilic antioxidant characteristics that decrease oxidative stress^(19)^.

Reports evaluating the anti-inflammatory properties of HSP-70 have found that it decreases the release of pro-inflammatory factors such as nuclear factor kappa B, matrix metalloproteinases, and reactive oxygen species and that it can prevent responses to inflammatory cytokines such as TNF-α and IL-1^(20)^.

The level of periostin is normally low in most adult tissues. The expressions of TGF-β and/or IL-4 and IL-13 are induced in macrophages and neutrophils in response to inflammation and in other types of cells in response to mechanical stress. These cytokines trigger the expression of periostin^(21)^. Periostin can sustain or amplify inflammatory responses in pathological conditions. However, it is secreted as a latent consequence of inflammatory responses rather than a regulating factor of the response^(22)^.

Mazur-Bialy et al. found that a high concentration of irisin significantly decreases the toll-like receptor 4 protein levels and the phosphorylation of nuclear factor-κB (NF-κB). Consequently, crucial pro-inflammatory cytokines as interleukin 1β, tumor necrosis factor α, interleukin 6, keratinocyte chemoattractant, monocyte chemotactic protein 1, and high mobility group box 1 reduce^(23)^. Irisin has been shown to alleviate oxidative stress by reducing the production of superoxide, peroxynitrite, and inducible nitric oxide synthase and by increasing production of antioxidant enzymes, including glutathione peroxidase, catalase, and superoxide dismutase^(24)^.

We are aware of the limitations of our study. We had to study HSP-70, periostin, and irisin in diluted aqueous humor samples because taking large aqueous humor samples would have been dangerous for our patients and contrary to ethical rules. In addition, we also applied strict exclusion criteria, and as a result, our population was not very large.

In conclusion, HSP-70, periostin, and irisin increase in the aqueous humor of patients with PEX without glaucoma. Further detailed studies are needed to determine the exact pathophysiologic roles of these substances in patients with PEX.
